# Genetic polymorphisms of 54 mitochondrial DNA SNP loci in Chinese Xibe ethnic minority group

**DOI:** 10.1038/srep44407

**Published:** 2017-03-22

**Authors:** Chun-Mei Shen, Li Hu, Chun-Hua Yang, Cai-Yong Yin, Zhi-Dan Li, Hao-Tian Meng, Yu-Xin Guo, Ting Mei, Feng Chen, Bo-Feng Zhu

**Affiliations:** 1 Key Laboratory of Shaanxi Province for Craniofacial Precision Medicine Research, College of Stomatology, Xi’an Jiaotong University, Xi’an, Shaanxi, 710004, P. R. China; 2 Clinical Reaserch Center of Shaanxi Province for Dental and Maxillofacial diseases, College of Stomatology, Xi’an Jiaotong University, Xi’an, Shaanxi, 710004, P. R. China; 3 Department of Forensic Genetics, School of Forensic Medicine, Southern Medical University, Guangzhou, Guangdong, 510515, P. R. China; 4 Institute of Brain and Behavioral Sciences, College of Life Sciences, Shaanxi Normal University, Xi’an, Shaanxi, 710062, P. R. China; 5 Department of Forensic Medicine, Nanjing Medical University, Nanjing, Jiangsu, 210029, P. R. China; 6 Department of Biochemistry and Molecular Biology, Basic Medicine College, Xinjiang Medical University, Urumqi, 830011, P. R. China

## Abstract

We analyzed the genetic polymorphisms of 54 mitochondrial DNA (mtDNA) variants in Chinese Xibe ethnic minority group. A total of 137 unrelated healthy volunteers from Chinese Xibe group were the objects of our study. Among the selected loci, there were 51 variable positions including transitions and transversions, and single nucleotide transitions were common (83.93%) versus transversions. These variations defined 64 different mtDNA haplotypes exclusive of (CA)_n_ and 9 bp deletion variation. The haplotype diversity and discrimination power in Xibe population were 0.9800 ± 0.004 and 0.9699, respectively. Besides, we compared Xibe group with 18 other populations and reconstructed a phylogenetic tree using Neighbor-Joining method. The result revealed that Xibe group was a close to Xinjiang Han and Yanbian Korean groups. Our data also indicated that Xibe group has a close relationship with Daur and Ewenki groups, which is reflected by the history that Xibe was influenced by Daur and Ewenki groups during the development of these groups. In conclusion, the variants we studied are polymorphic and could be used as informative genetic markers for forensic and population genetic application.

Genetic analysis of human mitochondrial DNA (mtDNA) is an important and indispensable part in population genetic studies all over the world. MtDNA hypervariable segments is an advanced research hotspot due to their molecular properties, inheritance pattern, specific population polymorphism, rapid evolutionary rate, low recombination rate and so on^1^,^2^,^3^,^4^,^5^,^6^. Meanwhile, frequencies of certain mtDNA sequence variations in a given population play an important role in the field of forensic genetics. The biomaterials from crime scenes are affected by various factors, such as human activities, environment, long intervals after the crime and so on, the analysis of mtDNA provides a more critical and helpful approach to specimen source identification on highly degraded biomaterials including teeth and dated bloodstains that contain infinitesimal nuclear DNA than usual^7^,^8^. Hence, the genetic analysis of mtDNA is utilized widely for the forensic purposes, especially for maternal lineage study and parentage identification. In addition, mtDNA sequence types as available mitochondrial markers are strongly correlated with geographic origin and benefit anthropological researches^9^,^10^.

Xibe as an ethnic minority group, is officially recognized by China. It had a population of 190,481 according to the latest China population census in 2010. The Xibe people mostly live in Jilin (bordering North Korea), Xinjiang and Liaoning. The Xibe people in northeast (Jilin and Liaoning provinces) and northwest (Xinjiang Uygur Autonomous Region) China have obvious characteristics due to the different geographical locations. Chinese is the first language of northeastern Xibe people while people from Xinjiang Xibe group speak a southern language which is a mixture of Chinese and Xinjiang languages including Kazakh, Russian and Uygur^11^. In recent years, many studies focusing on different autosomal short tandem repeat (STR) loci of Xibe group have been reported^12^,^13^,^14^. In this study, we collected the data of 54 mtDNA variants in Xibe ethnic group in order to study genetic polymorphisms in the field of forensic science and infer the genetic relationships between Xibe and other groups.

## Materials and Methods

### Samples

In total, 137 blood samples were collected from unrelated healthy donors of Xibe ethnic group in Ili, Xinjiang Uygur Autonomous region. The volunteers were randomly selected from the Xibe group and their ancestors must have been living there for at least three generations. Every participant wrote informed consent and blood samples were obtained respectively according to standard procedures. The study was conducted in accordance with the human and ethical research principles of Xi’an Jiaotong University Health Science Center, China and approved by the ethics committee of Xi’an Jiaotong University Health Science Center.

### DNA extraction, amplification and genotyping

DNA was extracted using the Chelex-100 as described in previous protocol^15^. Multiplexed PCR amplifications of 60 variants were co-amplified in 5 fluorescence-based reaction using Expressmarker mtDNA-SNP 60 kit (nt10398, 9 bp, 10873, 3010, 709, 7196, 12705, 3970, 13104, 10310, 5178, 13928, 6446, 8414, 8793, 8794, 15043, 16311, 16126, 16129, 8701, 8697, 4883, 10400, CA, 1719, 14668, 12811, 9824, 9123, 7028, 11719, 8584, 11251, 8020, 5460, 2706, 11215, 4216, 12372, 16362, 9698, 1541, 8684, 9477, 4491, 1811, 16316, 16319, 9545, 152, 14569, 8964, 10397, 3348, 4833, 7600, 5417, 5442, 15784, AGCU ScienTech Incorporation, Jiangsu, Wuxi). Briefly, PCR amplification system (25 μl) contained 1 μl genomic DNA, 10 μl reaction mix, 5 μl primers, 1μl tag DNA polymerase (5U) and 8 μl sdH_2_O. The cycling parameters were set up according to the manufacturer’s protocol. The PCR production of 1 μl was mixed with 0.5 μl AGCUMarkerSIZ-500 and 12 μl Hi-Di formamide. Electrophoresis was performed by the ABI Prism 3130XL Genetic analyzer and fragment sizing was analyzed by GeneMapper ID v3.2.1 software (Applied Biosystems, USA). Control DNA 9947A was used as the positive control in our experiment.

### Quality control

The multiplex allele-specific system was based on allele-specific primers which can analyze single nucleotide polymorphism (SNP) effectively. The 3′ end of the specific primers with different length were matched to the corresponding allele. In order to avoid non-specific extension, mismatched base was introduced into the third or another position at 3′ end of the specific primers. Each common primer was labeled with one fluorescence (FAM, HEX or TAMRA in our study) for detection, respectively. Allelic ladder (known allele of human) and positive control were used in our study for comparison and typing. The PCR production was mixed with a marker (DNA size was known) for the determination of DNA size (primers with fluorescence, allelic ladder, positive control 9947A and the marker were provided in the kit). Furthermore, negative control and reagent blank extraction experiments were also carried out in our study. All experimental procedures were operated exactly according to manufacturer’s instruction and laboratory internal standard to minimize errors.

### Statistical analysis

However, this kit has a certain detection rate and the rate is affected by many factors: the experience of experimenters, the technology of detection, the quality of samples, the sensitivity of primers and so on. Due to technology problem and genetic variation of these markers in Xibe populations, 4 loci did not generated clear genotypic results in all Xibe individuals. Moreover, (CA)n and 9 bp variants were not widely studied in different groups. Therefore we took the data of these 54 variants for further analysis (nt152, 709, 1541, 1719, 1811, 2706, 3010, 3348, 3970, 4216, 4491, 4833, 4883, 5178, 5417, 5442, 5460, 6446, 7028, 7196, 7600, 8020, 8414, 8584, 8684, 8964, 9123, 9477, 9545, 9698, 9824, 10310, 10397, 10398, 10400, 10873, 11215, 11251, 11719, 12372, 12705, 12811, 13104, 13928, 14569, 14668, 15043, 15784, 16126, 16129, 16311, 16316, 16319, 16362). The sequence data about 54 mtDNA variants of Xibe group were aligned with the revised Anderson Reference Sequence (rCRS) of the Human mitochondrial DNA for statistical analysis^16^,^17^. The software DNAsp 5.0 and Mega 4.0 were employed to estimate the gene diversities and other genetic parameters in Chinese Xibe group. Haplotype diversity^18^ is a measure of the uniqueness of a particular haplotype in a certain population. The haplotype diversity (H) is computed as:


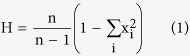


Where x_i_ is the (relative) haplotype frequency of each haplotype in the sample and n is the sample size. Nucleotide diversity (π)^19^ is the average number of nucleotide differences per site between two DNA sequences selected randomly from a given population. Where x_i_ and x_j_ are the frequencies of the i-th and the j-th sequence, respectively. π_ij_ is the number of nucleotide differences per nucleotide site between the i-th and the j-th sequences and n is the number of sample.


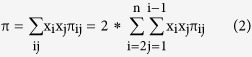


The probability of two random individuals from a population having the same mtDNA types was calculated as P = ∑x_i_^2^ and the discrimination power is 1 − ∑x_i_^2^ (x_i_ is the frequency of the i-th mtDNA haplotype) for evaluating the probability of two unrelated random samples having different phenotypes within a certain population.

Besides, we used Arlequin3.0 to calculate the *Fst* values among Xibe ethnic group and other groups using the 54 shared variants based on pair-wise comparisons (nt152, nt16126, nt16129, nt16311, nt16316, nt16319, nt16362 were in the control region and the remaining were in the coding region). On this basis, principle component analysis (PCA) and phylogenetic tree reconstruction were operated by SPSS19.0 and Mega 4.0 respectively to study the Xibe genetic background. *P* values below 0.05 were considered to be statistically significant.

## Results

### Genetic parameters analysis

The 54 selected mtDNA variants were successfully detected in all samples of the Chinese Xibe ethnic minority group, and the allele frequencies were showed in Table 1 while other 6 variants (nt8697, 8701, 8793, 8794, (CA)n and 9 bp) were excluded for vaguely genotypic results (nt8697, 8701, 8793 and 8794) or localized application ((CA)n and 9 bp). The mutation rates among Xibe ethnic group and other groups^20^,^21^,^22^,^23^,^24^,^25^,^26^ were listed in Supplemental Table 1(S1). In the studied group, single nucleotide transitions were the most common polymorphism (83.93%). At nt5178, nt7196 and nt13928 variants, the polymorphisms were caused by transversion. At the locus nt9824, transition and transversion were existed simultaneously (A/T/C), and there were no polymorphisms at nt3348, nt4491, nt6446, nt8684 and nt13104 variants.

Generally, the relative frequency of the mtDNA variants between 0.20~0.80 is considered to be polymorphic SNPs^27^. Moreover, as shown in S1, the variants 709 and 14569 were specific to Xibe compared to Guangdong Han^20^, Liaoning Han^24^, Qingdao Han^24^, Xinjiang Han^24^, Wuhan Han^24^ and Yunnan Han^24^ groups. Similarly, the variants 14668 and 15043 mutations were observed in Xibe group rather than Daur^21^, Mongolian^21^, Korean^21^ and Ewenki^21^ groups. Therefore, these loci could be highly polymorphic and helpful in the forensic application in Xibe ethnic group.

As mentioned above, there were 51 variable positions (transition and transversion) in the studied Xibe group, and these variations defined 64 different mtDNA haplotypes (excluding (CA)_n_ and 9 bp deletion) that were listed in Table 2. In addition, the two variable sites ((CA)_n_ and 9 bp deletion) defined two additional haplotypes as shown in Table 2. Which means, 61-a and 61-b will be the same haplotype if (CA)_n_ and 9 bp deletion are not taken into account. The differences between them are the repeat number of (CA) which is 5 in 61-a while 4 in 61-b.

As shown in Table 2, the haplotype that is comprised of the variants including 2706G, 3010A, 4883T, 5178A, 7028T, 8414T, 10398G, 10400T, 10873C, 11719A, 12705T, 14668T, 15043A and 16362C (No. 61, 15/137) was the most common haplotype among all 64 haplotypes. Likewise, No. 61-a haplotype (variants: 2706G, 3010A, 4883T, 5178A, 7028T, 8414T, 10398G, 10400T, 10873C, 11719A, 12705T, 14668T, 15043A and 16362C, the number of (CA) repeats was 5, the 9 bp was NORM) was the most frequent (12/137) in our study when (CA)_n_ and 9 bp deletion were taken into consideration. However, unique haplotypes which were only observed in a single person accounted for 27% (37/137) of the total subjects. Besides, the shared haplotypes were listed in Supplemental Table 2(S2) which included the haplotypes found between Xibe and other groups or the haplotype whose frequency in Xibe group was higher than 0.02. All the listed haplotypes were specific to Xibe group except for No. 14, No. 17, No. 30, No. 31, No. 32 and No. 61 (the sequence number was consistent with Table 2). No. 61 was the most common haplotype in Xibe ethnic group while lower frequency or none was observed in the rest groups. Table 3 shows the genetic parameters of the studied mtDNA variants in the Chinese Xibe group. The haplotype diversity and the nucleotide diversity in the studied Xibe population were 0.9800 ± 0.004 and 0.1875 respectively. The DP value in Chinese Xibe samples was 0.9699.

### Principle component analysis

**Figure 1:**
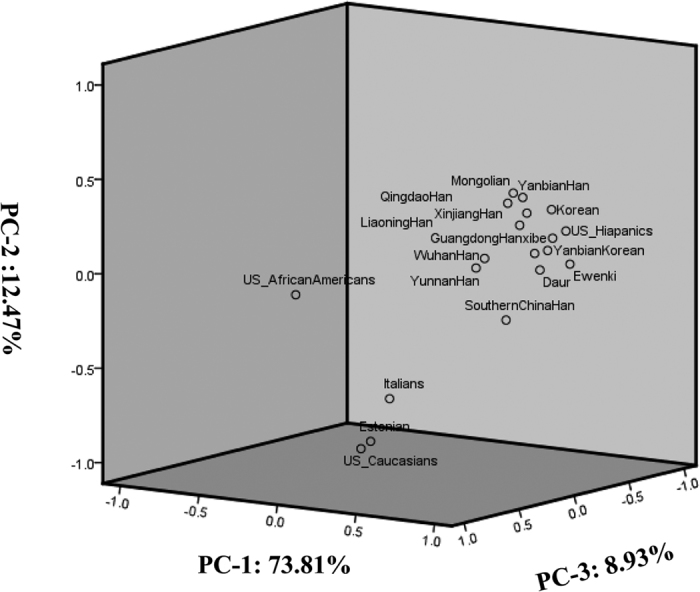
The distribution of different groups analyzed by principle component based on the *Fst* values between Xibe and 18 other groups.

*Fst* and *p* values between Xibe and 18 other groups^20^,^21^,^22^,^23^,^24^,^25^,^26^ were showed in Table 4, and data below the diagonal were *Fst* values, while the above data were *p* values. The PCA plot based on *Fst* values was showed in Fig. 1 and Supplemental Figs 1–3. All groups had a significant difference with Hispanics and African Americans. Among the six Chinese Han groups (Qingdao Han, Wuhan Han, Guangdong Han, Liaoning Han, Yunnan Han and Xinjiang Han groups), no difference was observed between each other except for the comparisons between Wuhan Han and Guangdong Han, Xinjiang Han and Yunnan Han groups. The results also demonstrated the relationships between Xibe and other groups. According to the results, Xibe group was the closest to Xinjiang Han group (*Fst* = 0.00276, *p* > 0.05) while the farthest to Italians (*Fst* = 0.14807, *p* < 0.05). Meanwhile, the Xibe group also had a relatively close genetic distance to Guangdong Han, Yanbian Korean, Liaoning Han and Daur groups. Similarly, the same results were shown by the PCA plot. Xibe, Guangdong Han, Liaoning Han and Yanbian Korean groups were in one cluster. In contrast, African Americans, Italians, Estonians and Caucasians were dispersed in the plot.

### Phylogenetic analysis

**Figure 2:**
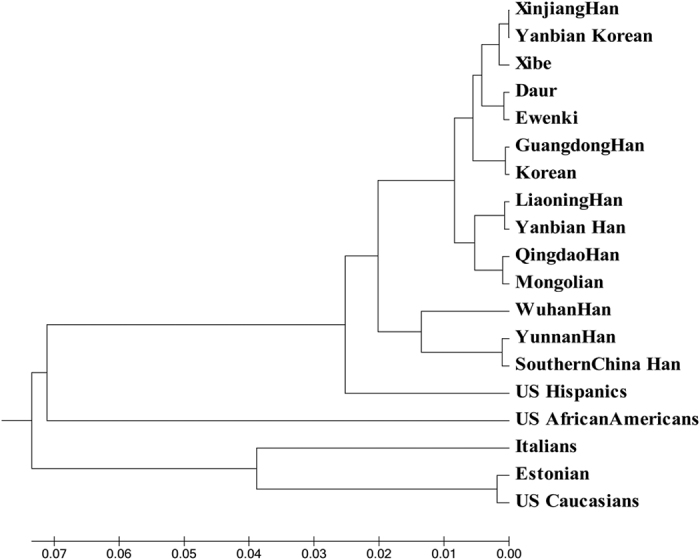
The phylogenetic tree was reconstructed by the neighboring-joining method.

In the phylogenetic tree based on Xibe and 18 other groups (Fig. 2), Italians, Estonians and Caucasians were in one cluster, the remaining 16 groups were in another. As mentioned above, Xibe, Yanbian Korean and Xinjiang Han had closer distance between each other and they were distributed in the same branch. Moreover, among these five groups (Xibe, Yanbian Korean, Xinjiang Han, Daur and Ewenki) that were located in the same sub branch, Daur and Ewenki were on a separated branch to the other three groups. It reveals that Xibe group was also close to Daur and Ewenki groups. As shown in Fig. 2, Liaoning Han, Yanbian Han, Qingdao Han and Mongolian groups constructed a sub branch, which meant these populations have a closer genetic relationship with each other in a way.

## Discussion

Mitochondrial DNA analysis is the only effective method that can be utilized for the bio-materials containing infinitesimal nuclear DNA^7^,^8^. The analysis of mtDNA has an irreplaceable value in forensic application for its unique characteristics such as strong sensitivity, maternal genetic characteristics and so on. Hence, the analysis of mtDNA is competent for specimen source identification as well as excluding the innocent.

The analysis of the relative frequency could reveal some unique characteristic features in Chinese Xibe group due to some variants observed with high polymorphisms while some with no polymorphisms. Due to different specific primers, experimental principles, operation methods and reaction conditions, we could not distinguish the complicated polymorphism caused by substitution, insertion and deletion. But through the analysis of these polymorphic variants in Xibe group, we can still find accurately the criminal from suspects and improve the discrimination power in forensic cases. Meanwhile, haplotypes of mtDNA variants can be used as a new genetic marker for personal identification in the Chinese Xibe group. In addition, (CA)_n_ is a short tandem repeat (STR) marker with a dinucleotide repeat (C and A) in mitochondrial D-loop region (from nt00514 to nt00524 in the rCRS). The repeat number is of polymorphism, lower than 10 normally and has some certain relationship with tumorigensis according to the previous report^28^. As well, 9 bp sequence deletion is also a genetic marker in mtDNA. Its geographical distribution was correlated with the trace of human immigration. So the analysis of (CA)_n_ and 9 bp deletion can improve the forensic power of SNPs and haplotypes.

The results of *Fst*, PCA plot and phylogenetic tree simultaneously pointed out that our studied Xibe group had nearer genetic relationships with Xinjiang Han, Yanbian Korean and Daur groups. The branch that was composed of Xibe, Xinjiang Han and Yanbian Korean supported the opinion of nationwide territorial dispersion of Xibe people during Jin Dynasty. Furthermore, the closer relationship between Xibe, Daur and Ewenki groups(from Inner Mongolia^21^) which was confirmed by the previous report showed the relocation of Xibe people in 1700A.D^14^. Besides, the Xibe group was incorporated by Daur due to the fact that Khorchin dedicated them to Kangxi Emperor in exchange for silver in 1962. Besides, intermarriage between different races existed inevitably during abovementioned historical events. Hence, we inferred that the Xibe ethnic group probably had common ancestors with Xinjiang Han and Yanbian Korean groups. In addition, Daur and Ewenki groups also had high possibilities to own an ancestor with Xibe group or influence Xibe greatly during the development of these groups.

## Concluding remarks

The selected 54 mtDNA variants were utilized for analyzing the genetic polymorphism of the Xibe ethnic minority group from China. Among these variants, a total of 14 mtDNA SNP loci (nt152, nt709, nt3010, nt4883, nt5178, nt8414, nt10398, nt10400, nt10873, nt12705, nt14668, nt15043, nt16129, and nt16362) were found to be genetic polymorphic and can be used as valid genetic markers for forensic and population genetic application. Meanwhile, 51 variable positions (transition and transversion) defined 64 different mtDNA haplotypes (excluding (CA)_n_ and 9 bp deletion). The haplotype diversity was 0.9800 ± 0.004. Finally, we made a comparison between Xibe group and other populations to infer the genetic structure and relationships. The results of *Fst*, PCA and phylogenetic analysis showed that Xibe group had closer genetic relationships with Xinjiang Han and Yanbian Korean. However, more details should be collected to reconstruct the phylogenetic tree and research the migration and evolution of human populations.

## Additional Information

**How to cite this article:** Shen, C.-M. *et al*. Genetic polymorphisms of 54 mitochondrial DNA SNP loci in Chinese Xibe ethnic minority group. *Sci. Rep.* **7**, 44407; doi: 10.1038/srep44407 (2017).

**Publisher's note:** Springer Nature remains neutral with regard to jurisdictional claims in published maps and institutional affiliations.

## Supplementary Material

Supplementary Information

## Figures and Tables

**Table 1:** The allele frequencies of 54 mtDNA SNP loci in the Chinese Xibe ethnic minority group.

Loci	Allele	Frequency
152	T/C	0.78/0.22
709	G/A	0.77/0.23
1541	T/C	0.99/0.01
1719	G/A	0.98/0.02
1811	A/G	0.99/0.01
2706	G/A	0.93/0.07
3010	G/A	0.73/0.27
3348	A	1
3970	C/T	0.85/0.15
4216	T/C	0.99/0.01
4491	G	1
4833	A/G	0.98/0.02
4883	C/T	0.70/0.30
5178	C/A	0.70/0.30
5417	G/A	0.96/0.04
5442	T/C	0.97/0.03
5460	G/A	0.97/0.03
6446	G	1
7028	T/C	0.94/0.06
7196	C/A	0.85/0.15
7600	G/A	0.99/0.01
8020	G/A	0.91/0.09
8414	C/T	0.74/0.26
8584	G/A	0.80/0.20
8684	C	1
8964	C/T	0.94/0.06
9123	G/A	0.98/0.02
9477	G/A	0.99/0.01
9545	A/G	0.91/0.09
9698	T/C	0.99/0.01
9824	T/A/C	0.88/0.07/0.05
10310	G/A	0.86/0.14
10397	A/G	0.96/0.04
10398	G/A	0.64/0.36
10400	T/C	0.55/0.45
10873	C/T	0.55/0.45
11215	C/T	0.97/0.03
11251	A/G	0.99/0.01
11719	A/G	0.94/0.06
12372	G/A	0.93/0.07
12705	T/C	0.64/0.36
12811	T/C	0.98/0.02
13104	A	1
13928	G/C	0.85/0.15
14569	G/A	0.97/0.03
14668	C/T	0.74/0.26
15043	A/G	0.55/0.45
15784	T/C	0.95/0.05
16126	T/C	0.96/0.04
16129	G/A	0.77/0.23
16311	T/C	0.89/0.11
16316	A/G	0.99/0.01
16319	G/A	0.93/0.07
16362	T/C	0.61/0.39

**Table 2:** Haplotypes and variant positions in the Chinese Xibe ethnic group according to the revised Cambridge Reference Sequence (rCRS).

Haplotypes	Number	Variants	9 bp	(CA)n
1	6	2706G, 7028T, 7196A, 8584A, 9545G, 10398G, 10400T, 10873C, 11719A, 12705T, 15043A	NORM	5
2	5	152C, 709A, 2706G, 3970T, 7028T, 10310A, 11719A, 13928C, 16129A	NORM	4
3	5	2706G, 5417A, 7028T, 11719A, 12372A, 12705T, 16129A	NORM	5
4	4	2706G, 3970T, 7028T, 10310A, 11719A, 13928C, 16311C	NORM	4
5	4	2706G, 3970T, 7028T, 10310A, 11719A, 13928C, 16129A	NORM	4
6	4	2706G, 3970T, 7028T, 10310A, 11719A, 13928C	NORM	5
7	4	152C, 2706G, 3010A, 4883T, 5178A, 7028T, 8020A, 8414T, 8964T, 9824A, 10398G, 10400T, 10873C, 11719A, 12705T, 14668T, 15043A, 16362C	NORM	4
8	4	2706G, 7028T, 7196A, 8584A, 9545G, 10398G, 10400T, 10873C, 11719A, 12705T, 15043A, 16129A	NORM	5
9	3	709A, 2706G, 7028T, 8584A, 10398G, 11719A, 16362C	DEL	4
10	3	2706G, 3010A, 4883T, 5178A, 7028T, 8020A, 8414T, 8964T, 9824A, 10398G, 10400T, 10873C, 11719A, 12705T, 14668T, 15043A, 16362C	NORM	4
11	3	2706G, 4883T, 5178A, 7028T, 10397G, 10398G, 10400T, 10873C, 11719A, 12705T, 15043A, 16362C	NORM	4
12	3	2706G, 5460A, 7028T, 9824C, 10398G, 10400T, 10873C, 11719A, 12705T, 12811C, 15043A, 16129A	NORM	5
13	3	2706G, 5442C, 7028T, 9824C, 10398G, 10400T, 10873C, 11719A, 12705T, 15043A, 16319A	NORM	4
14	3	**rCRS**	NORM	5
15	2	709A, 2706G, 7028T, 9123A, 11719A	DEL	4
16	2	2706G, 7028T, 11719A, 16129A, 16311C	DEL	4
17	2	709A, 2706G, 4216C, 7028T, 11251G, 11719A, 16126C	NORM	5
18	2	16126C	NORM	5
19	2	152C, 16362C	NORM	4
20	2	1541C, 2706G, 3970T, 7028T, 11719A, 13928C	NORM	5
21	2	152C, 1719A, 2706G, 3010A, 4883T, 5178A, 7028T, 8414T, 10398G, 10400T, 10873C, 11719A, 12705T, 14668T, 15043A, 16311C, 16362C	NORM	5
22	2	152C, 709A, 2706G, 7028T, 7196A, 8584A, 10398G, 10400T, 10873C, 11719A, 12705T, 15043A, 15784C	NORM	5
23	2	709A, 2706G, 7028T, 10398G, 10400T, 10873C, 11719A, 12705T, 16311C	NORM	5
24	2	152C, 2706G, 7028T, 7196A, 8584A, 10398G, 10400T, 10873C, 11719A, 12705T, 15043A, 15784C, 16129A	NORM	5
25	2	152C, 2706G, 7028T, 7196A, 8584A, 10398G, 10400T, 10873C, 11719A, 12705T, 15043A, 15784C	NORM	5
26	2	152C, 2706G, 7028T, 7196A, 8584A, 9545G, 10398G, 10400T, 10873C, 11719A, 12705T, 15043A	NORM	5
27	1	709A, 2706G, 7028T, 10310A, 11719A	DEL	5
28	1	2706G, 7028T, 9123A, 11719A	DEL	4
29	1	2706G, 7028T, 11719A	DEL	5
30	1	3010A	NORM	5
31	1	1811G, 2706G, 7028T, 11719A, 12372A	NORM	5
32	1	2706G, 7028T, 9477A, 11719A, 12372A	NORM	8
33	1	152C, 2706G, 3970T, 7028T, 8020A, 10310A, 11719A, 13928C, 16311C	NORM	5
34	1	2706G, 3970T, 7028T, 11719A, 13928C	NORM	5
35	1	2706G, 7028T, 11719A, 12705T, 16126C, 16311C, 16319A	NORM	5
36	1	2706G, 7028T, 11719A, 12705T, 16311C, 16319A	NORM	4
37	1	152C, 7028T, 11719A, 12705T, 16319A, 16362C	NORM	4
38	1	152C, 2706G, 7028T, 11719A, 12705T, 16319A, 16362C	NORM	4
39	1	152C, 2706G, 7028T, 11719A, 12705T, 16319A	NORM	4
40	1	709A, 2706G, 3010A, 4883T, 5178A, 7028T, 8020A, 8414T, 8964T, 9824A, 10398G, 10400T, 10873C, 11719A, 12705T, 14668T, 15043A, 16362C	NORM	4
41	1	152C, 709A, 2706G, 3010A, 4883T, 5178A, 7028T, 8414T, 10398G, 10400T, 10873C, 11719A, 12705T, 14668T, 15043A, 16362C	NORM	5
42	1	152C, 2706G, 3010A, 4883T, 5178A, 7028T, 8414T, 10398G, 10400T, 10873C, 11719A, 12705T, 14668T, 15043A, 16311C, 16362C	NORM	5
43	1	2706G, 3010A, 4883T, 5178A, 7028T, 8020A, 8414T, 9824A, 10398G, 10400T, 10873C, 11719A, 12705T, 14668T, 15043A, 16362C	NORM	4
44	1	3010A, 4883T, 5178A, 7028T, 8414T, 10398G, 10400T, 10873C, 11719A, 12705T, 14668T, 15043A, 16362C	NORM	4
45	1	2706G, 3010A, 4883T, 5178A, 7028T, 8414T, 10398G, 10400T, 10873C, 11215T, 11719A, 12705T, 14668T, 15043A, 16362C	NORM	5
46	1	2706G, 3010A, 4883T, 5178A, 7028T, 8414T, 10398G, 10400T, 10873C, 11719A, 12705T, 14668T, 15043A16316G, 16362C	NORM	7
47	1	709A, 2706G, 7028T, 7196A, 8584A, 9545G, 10398G, 10400T, 10873C, 11719A, 12705T, 15043A, 16129A	NORM	5
48	1	709A, 2706G, 4883T, 5178A, 7028T, 10397G, 10398G, 10400T, 10873C, 11719A, 12705T, 15043A, 16362C	NORM	5
49	1	152C, 709A, 2706G, 7028T, 10398G, 10400T, 10873C, 11719A, 12705T, 15043A, 16311C	NORM	5
50	1	709A, 2706G, 4833G, 5460A, 7028T, 10398G, 10400T, 10873C, 11719A, 12705T, 14569A, 15043A, 16362C	NORM	5
51	1	709A, 2706G, 4833G, 7028T, 7600A, 9477A, 10398G, 10400T, 10873C, 11719A, 12705T, 14569A, 15043A, 16362C	NORM	5
52	1	709A, 2706G, 4833G, 7028T, 7600A, 10398G, 10400T, 10873C, 11719A, 12705T, 14569A, 15043A, 16362C	NORM	5
53	1	709A, 2706G, 7028T, 10398G, 10400T, 10873C, 11719A, 12705T, 14569A, 15043A, 16362C	NORM	6
54	1	152C, 2706G, 7028T, 7196A, 8584A, 10398G, 10400T, 10873C, 11719A, 12705T, 15043A, 15784C, 16126C	NORM	5
55	1	152C, 2706G, 4883T, 5178A, 7028T, 10397G, 10398G, 10400T, 10873C, 11719A, 12705T, 15043A, 16362C	NORM	5
56	1	2706G, 5442C, 7028T, 9824C, 10398G, 10400T, 10873C, 11719A, 12705T, 15043A, 16362C	NORM	4
57	1	2706G, 7028T, 10398G, 10400T, 10873C, 11719A, 12705T, 15043A	NORM	5
58	1	1811G, 2706G, 7028T, 9698C, 10398G, 11719A, 12372A	NORM	4
59	1	1719A, 2706G, 7028T, 10398G, 11719A, 12705T, 15043A, 16129A	NORM	5
60	1	2706G, 5417A, 7028T, 10398G, 11719A, 12372A, 12705T, 16129A	NORM	4
61-a	12	2706G, 3010A, 4883T, 5178A, 7028T, 8414T, 10398G, 10400T, 10873C, 11719A, 12705T, 14668T, 15043A, 16362C	NORM	5
61-b	3	2706G, 3010A, 4883T, 5178A, 7028T, 8414T, 10398G, 10400T, 10873C, 11719A, 12705T, 14668T, 15043A, 16362C	NORM	4
62-a	3	709A, 2706G, 7028T, 8584A, 10398G, 11719A	DEL	4
62-b	2	709A, 2706G, 7028T, 8584A, 10398G, 11719A	DEL	5
63-a	2	2706G, 3010A, 4883T, 5178A, 7028T, 8414T, 10398G, 10400T, 10873C, 11215T, 11719A, 12705T, 14668T, 15043A, 16129A, 16362C	NORM	5
63-b	1	2706G, 3010A, 4883T, 5178A, 7028T, 8414T, 10398G, 10400T, 10873C, 11215T, 11719A, 12705T, 14668T, 15043A, 16129A, 16362C	NORM	6
64-a	1	2706G, 3010A, 4883T, 5178A, 7028T, 8020A, 8414T, 10398G, 10400T, 10873C, 11719A, 12705T, 14668T, 15043A, 16319A, 16362C	NORM	4
64-b	1	2706G, 3010A, 4883T, 5178A, 7028T, 8020A, 8414T, 10398G, 10400T, 10873C, 11719A, 12705T, 14668T, 15043A, 16319A, 16362C	DEL	4

Sequence data was aligned with the Revised Cambridge Reference Sequence (rCRS). The mtDNA sequences that have no mutation in Xibe ethnic group compared with the rCRS were labeled as rCRS. Suffixes A, G, C and T represent the observed differences to rCRS. NORM: the deletion of 9bp (CCCCCTCTA), DEL: non- deletion of 9bp. (CA)_n_: the number of (CA) repeats that are situated from nt00514 to nt00524 referred to the mtDNA genome according to the Revised Anderson Reference Sequence (rCRS). The haplotype –a/−b indicate different haplotypes based on different conditions of 9bp deletion and (CA)_n_.

**Table 3:** Genetic parameters in the Chinese Xibe group (excluding the (CA)_n_ and 9bp).

Indexes	Value
No. of haplotypes	64
No. of polymorphic sites	51
No. of indels	0
Haplotype diversity	0.9800 ± 0.004
Nucleotide diversity	0.1875
Random match probability (P)	0.0301
Discrimination power (DP)	0.9699

**Table 4:** *Fst* and *P* values between Xibe and 18 other groups.

Populations	1	2	3	4	5	6	7	8	9	10	11	12	13	14	15	16	17	18	19
1	*	0.85586	0.16216	0.34234	0.01802	0.44144	0.05405	0.00000	0.00000	0.00000	0.00000	0.61261	0.00000	0.10811	0.00901	0.83784	0.01802	0.08108	0.11712
2	0.01207	*	0.18919	0.20721	0.01802	0.27027	0.18018	0.00000	0.00901	0.00000	0.00000	0.80180	0.00000	0.06306	0.42342	0.58559	0.05405	0.33333	0.25225
3	0.00600	0.01253	*	0.67568	0.17117	0.89189	0.00901	0.00000	0.00000	0.00000	0.00000	0.30631	0.00000	0.29730	0.00000	0.21622	0.08108	0.07207	0.09910
4	0.00370	0.00967	0.00746	*	0.11712	0.90090	0.08108	0.00000	0.00000	0.00000	0.00000	0.32432	0.00000	0.81982	0.00000	0.15315	0.40541	0.13514	0.03604
5	**0.02953**	**0.04150**	0.01163	0.01640	*	0.15315	0.14414	0.00000	0.02703	0.00000	0.00000	0.03604	0.00000	0.01802	0.01802	0.04505	0.00000	0.00000	0.00000
6	0.00276	0.00622	0.01191	0.01465	0.01319	*	0.03604	0.00000	0.01802	0.00000	0.00000	0.43243	0.00000	0.92793	0.00000	0.49550	0.29730	0.32432	0.08108
7	0.01897	0.01111	**0.03971**	0.02478	0.01424	**0.03319**	*	0.00000	0.00000	0.00000	0.00000	0.07207	0.00000	0.00000	0.42342	0.10811	0.00901	0.00000	0.00000
8	**0.10341**	**0.12501**	**0.10719**	**0.16360**	**0.11345**	**0.13883**	**0.13693**	*	0.00000	0.00000	0.19820	0.00000	0.00000	0.00000	0.00000	0.00000	0.00000	0.00000	0.00000
9	**0.04803**	**0.05798**	**0.02868**	**0.04248**	**0.03615**	**0.02548**	**0.09566**	**0.14471**	*	0.00000	0.00000	0.01802	0.00000	0.00901	0.00000	0.00000	0.00000	0.03604	0.00000
10	**0.13039**	**0.15090**	**0.09262**	**0.10634**	**0.09568**	**0.12257**	**0.10556**	**0.15395**	**0.20950**	*	0.00000	0.00000	0.00000	0.00000	0.00000	0.00000	0.00000	0.00000	0.00000
11	**0.07079**	**0.08809**	**0.06769**	**0.10550**	**0.06741**	**0.08927**	**0.08290**	0.00370	**0.11759**	**0.09390**	*	0.00000	0.00000	0.00000	0.00000	0.00000	0.00000	0.00000	0.00000
12	0.00330	0.01551	0.00160	0.00388	**0.02759**	0.00007	0.02820	**0.12204**	**0.02451**	**0.15260**	**0.08956**	*	0.00000	0.11712	0.02703	0.32432	0.00000	0.65766	0.21622
13	**0.14807**	**0.23293**	**0.18028**	**0.23578**	**0.17993**	**0.20826**	**0.21701**	**0.06111**	**0.17269**	**0.27009**	**0.09414**	**0.18389**	*	0.00000	0.00000	0.00000	0.00000	0.00000	0.00000
14	0.01296	0.02628	0.00139	0.01109	**0.03937**	0.01497	**0.06095**	**0.18813**	**0.03761**	**0.14899**	**0.12947**	0.01596	**0.26063**	*	0.00000	0.13514	0.36036	0.17117	0.05405
15	**0.01956**	0.00226	**0.04947**	**0.05078**	**0.03974**	**0.05037**	0.00212	**0.10012**	**0.09713**	**0.14016**	**0.07384**	**0.02067**	**0.15662**	**0.08589**	*	0.05405	0.00000	0.00000	0.00000
16	0.00951	0.00913	0.00820	0.01160	**0.03557**	0.00234	0.02678	**0.09135**	**0.05386**	**0.12811**	**0.05317**	0.00447	**0.17365**	0.01548	0.02512	*	0.04505	0.05405	0.32432
17	**0.02346**	0.02467	0.02216	0.00195	**0.05666**	0.00363	**0.04190**	**0.22266**	**0.08261**	**0.15873**	**0.15665**	**0.03069**	**0.28530**	0.00153	**0.07600**	**0.03095**	*	0.04505	0.00000
18	0.01369	0.00114	0.01694	0.01092	**0.06220**	0.00609	**0.06427**	**0.19184**	**0.02947**	**0.20906**	**0.14997**	0.00629	**0.25551**	0.00986	**0.06233**	0.02596	**0.01785**	*	0.19820
19	0.01050	0.00383	0.01526	**0.03582**	**0.07232**	0.01602	**0.07454**	**0.10011**	**0.04560**	**0.18122**	**0.07716**	0.00712	**0.17245**	0.03326	**0.05567**	0.00152	**0.04837**	0.01241	*

Data with statistically significance were in bold.

1, Xibe; 2, Guangdong Han; 3, Liaoning Han; 4, Qingdao Han; 5, Wuhan Han; 6, Xinjiang Han; 7, Yuannan Han; 8, Estonian; 9, US_Hispanics; 10, US_African Americans.

11, US_Caucasians; 12, Yanbian Korean;13, Italians; 14, Yanbian Han; 15, Southern China Han; 16, Daur; 17, Mongolian; 18, Korean; 19, Ewenki.
