# Supplementary material for: Genetic polymorphisms of 54 mitochondrial DNA SNP loci in Chinese Xibe ethnic minority group
Source: Sci Rep. 2017 Mar 22;7:44407. doi: 10.1038/srep44407 (PMC5361124; doi:10.1038/srep44407)
Supplement: Supplementary Information [file srep44407-s1.pdf]

Genetic polymorphisms of 54 mitochondrial DNA SNP loci in Chinese Xibe ethnic minority group

Chun- Mei Shen[1,2,3,4*], Li Hu[5*], Chun-Hua Yang[2,3], Cai-Yong Yin[5], Zhi-Dan Li[2,3], Hao-Tian Meng[2,3], Yu- Xin Guo[2,3], Ting Mei[6], Feng Chen[5⊿], Bo-Feng Zhu[1,2,3⊿]

[1]*Department of Forensic Genetics, School of Forensic Medicine, Southern Medical University, Guangzhou, Guangdong 510515, P. R. China*

[2] *Key Laboratory of Shaanxi Province for Craniofacial Precision Medicine Research, College of Stomatology, Xi'an Jiaotong University, Xi'an, Shaanxi, 710004, P. R. China*

[3] *Clinical Reaserch Center of Shaanxi Province for Dental and Maxillofacial diseases, College of Stomatology, Xi'an Jiaotong University, Xi'an, Shaanxi, 710004, P. R. China*

[4]*Institute of Brain and Behavioral Sciences, College of Life Sciences, Shaanxi Normal University, Xi'an, Shaanxi 710062, P. R. China.*

[5]*Department of Forensic Medicine, Nanjing Medical University, Nanjing, Jiangsu 210029, P. R. China.*

[6] *Department of Biochemistry and Molecular Biology , Basic Medicine College, Xinjiang Medical University, Urumqi 830011, P. R. China*

Corresponding author: Prof. Bo-Feng Zhu, Department of Forensic Genetics, School of Forensic Medicine, Southern Medical University, Guangzhou, Guangdong 510515, P. R. China

E-mail:zhubofeng7372@126.com

*Both authors contributed equally to this work

⊿Both authors contributed equally to this work

Supplemental Figure 1. The distribution of different groups based on *Fst* analyzed by the first and the second principle component.

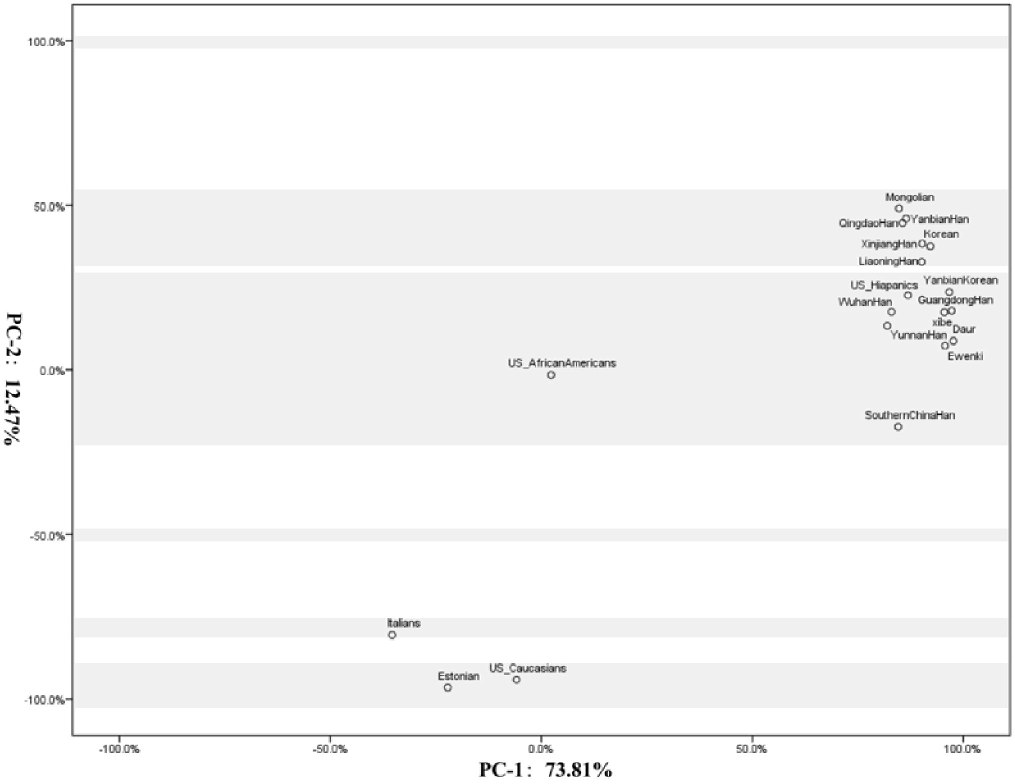

Supplemental Figure 2. The distribution of different groups based on *Fst* analyzed by the first and the third principle component.

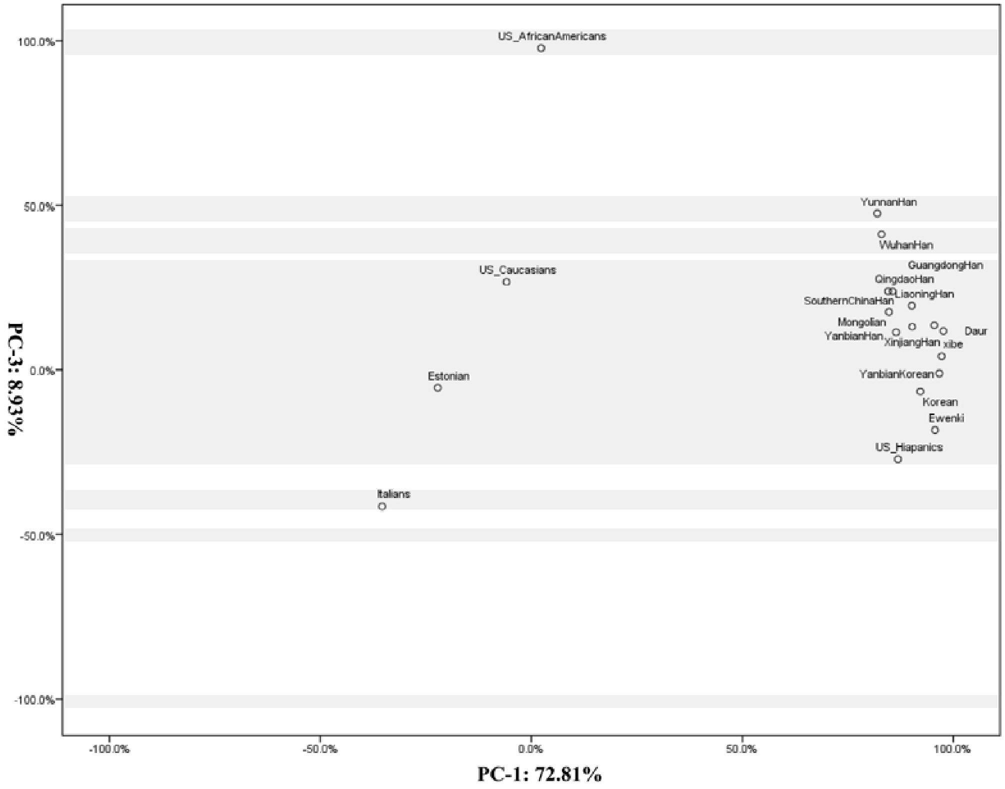

Supplemental Figure 3. The distribution of different groups based on *Fst* analyzed by the second and the third principle component.

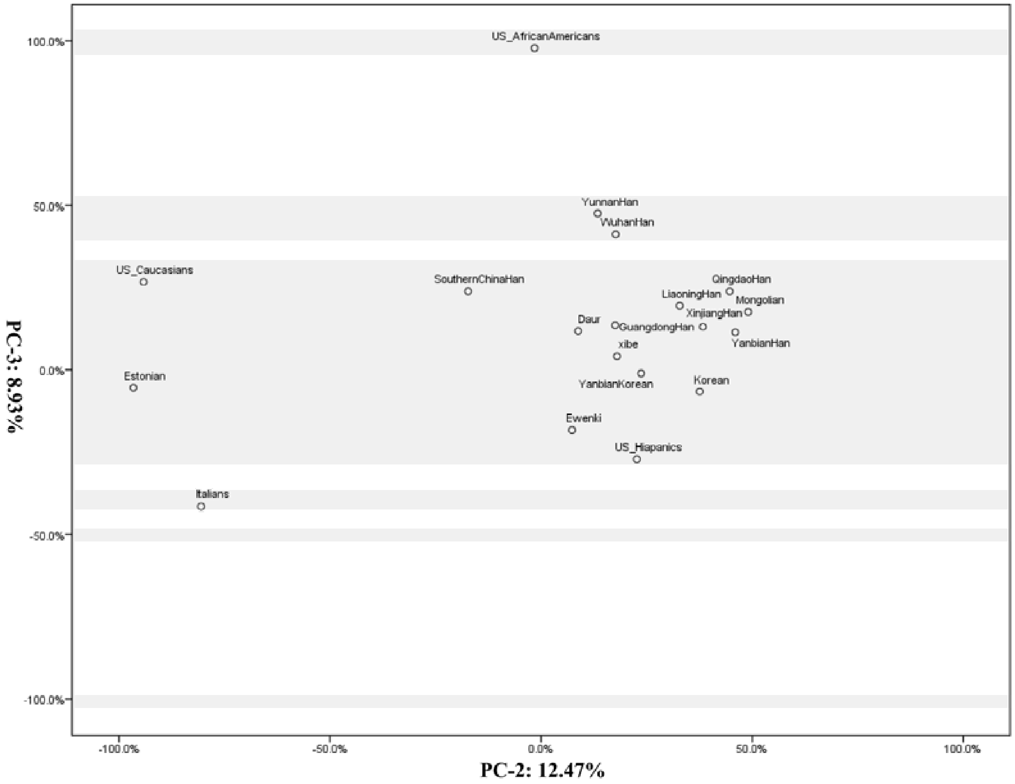

Supplemental Table 1.The mutation rates on shared loci in difference groups.

**Loci**	Xibe	GD	LN	QD	WH	XJ	YN	YK	YH	SC	DA	MG	KR	EK
**152**	0.22	0.13	0.29	0.34	0.31	0.3	0.26	0.15	0.33	0.13	0.22	0.33	0.13	0.11
**709**	**0.23**	**0**	**0**	**0**	**0**	**0**	**0**	0.13	*	*	*	*	*	*
**10310**	0.14	0.23	0.06	0.1	0.17	0.19	0.16	*	*	*	*	*	*	*
**10397**	0.04	0.06	0.06	0.1	0.05	0.06	0.05	*	*	*	*	*	*	*
**10398**	0.64	0.5	0.69	0.64	0.43	0.62	0.44	*	*	*	*	*	*	*
**10400**	0.55	0.5	0.63	0.6	0.38	0.55	0.42	*	*	*	*	*	*	*
**14569**	0.03	0	0	0	0	0	0	*	*	*	*	*	*	*
**14668**	**0.26**	*	*	*	*	*	*	*	*	*	**0**	**0**	**0**	**0**
**15043**	**0.55**	*	*	*	*	*	*	*	*	*	**0**	**0**	**0**	**0**
**15784**	0.05	*	*	*	*	*	*	0.02	*	*	0	0	0	0
**16126**	0.04	0	0.03	0.02	0	0.02	0.02	0.02	0.02	0	0.07	0.02	0	0.11
**16129**	0.23	0.27	0.13	0.24	0.24	0.17	0.42	0.22	0.16	0.35	0.18	0.31	0.21	0.11
**16311**	0.11	0.13	0.2	0.16	0.12	0.09	0.12	0.16	0.06	0.16	0.07	0.04	0.1	0.11
**16316**	0.01	0	0.06	0.02	0.05	0.04	0	0	0.02	0	0	0.13	0.04	0.09
**16319**	0.07	0.07	0.16	0.16	0.26	0.15	0.09	0.15	0.16	0.04	0.02	0.1	0.17	0.04
**16362**	0.39	0.4	0.39	0.46	0.21	0.43	0.26	0.42	0.51	0.25	0.36	0.54	0.54	0.43

Xibe (n=137): the studied ethnic group in our paper; GD (n=30): Guangdong Han; LN (n=51): Liaoning Han; QD (n=50): Qingdao Han; WH (n=42): Wuhan Han; XJ (n=47): Xinjiang Han; YN (n=43): Yunnan Han; YK (n=55): Yanbian Korean; YH (n=51): Yanbian Han; SC (n=69): Southern China Han; DA (n=45): Daur; MG (n=48): Mongolian; KR (n=48): Korean; EK (n=47): Ewenki. *: no sequence data. The mutation rates in bold were specific to Xibe group compared to other groups.

Supplemental Table 2. Different haplotypes of six groups.

Haplotype*(No.)	EST	HIS	AA	CAU	ITA	Xibe
30	0.1491	0.0442		0.1084	0.3103	0.0073
31	0.0526	0.0088				0.0073
32	0.0789	0.0265				0.0073
17	0.0439	0.0088				0.0146
14	0.1316	0.0354	0.0115	0.1084	0.4138	0.0219
9						0.0219
63						0.0219
10						0.0219
11						0.0219
12						0.0219
13						0.0219
4						0.0292
5						0.0292
6						0.0292
7						0.0292
8						0.0292
2						0.0365
3						0.0365
62						0.0365
1						0.0438
61		0.0088				0.1095

EST (n=114): Estonian; HIS (n=113): Hispanics; AA (n=87): African Americans; CAU (n=83): Caucasians; ITA (n=29): Italians; Xibe (n=137): the studied population in our paper. n: the number of population.

* all haplotypes were listed in the Supplemental Table 2 according to the following conditions (**No.**: the sequence number of haplotypes are consistent with Table 2): The shared haplotypes were listed between and the rest populations; the haplotype frequency of Xibe > 0.02.
